# Mimicking of Blood Flow Results in a Distinct Functional Phenotype in Human Non-Adherent Classical Monocytes

**DOI:** 10.3390/biology10080748

**Published:** 2021-08-04

**Authors:** Elisa Wirthgen, Melanie Hornschuh, Ida Maria Wrobel, Christian Manteuffel, Jan Däbritz

**Affiliations:** 1 Mucosal Immunology Group, Department of Pediatrics, Rostock University Medical Center, 18057 Rostock, Germany; melanie.hornschuh@med.uni-rostock.de (M.H.); jan.daebritz@med.uni-rostock.de (J.D.); 2 Department of Transfusion Medicine, Rostock University Medical Center, 18057 Rostock, Germany; IdaMaria.Wrobel@med.uni-rostock.de; 3 Institute of Behavioral Physiology, Leibniz Institute for Farm Animal Biology, 18196 Dummerstorf, Germany; christian.manteuffel@fbn-dummerstorf.de; 4 Center for Immunobiology, The Barts and the London School of Medicine and Dentistry, Blizard Institute, Barts Cancer Institute, Queen Mary University, London E1 4NS, UK

**Keywords:** classical monocytes, shear flow, GM-CSF, phenotype, cytokine release, cellular therapy

## Abstract

**Simple Summary:**

Monocytes are immune cells of increasing interest as cellular-based therapeutic products in inflammation-related diseases. The underlying mechanism is that isolated monocytes are modified outside the body. After re-injection, monocytes are recruited to the site of inflammation, exerting their therapeutic effect. One current challenge is that isolated monocytes rapidly lose migratory capacity during culture, limiting their therapeutic efficacy. During suspension culture, mimicking blood flow has been shown to preserve the migratory capacity. However, the effects on the inflammatory response and other functional properties have not been studied so far. Hence, the present study investigates the effect of shear flow on cytokine secretion and selected features of human blood-derived classical monocytes. Our results demonstrate that mimicking blood flow resulted in a distinct phenotype with an anti-inflammatory cytokine response and a higher migratory capacity than cultured under static conditions. These features could be particularly relevant for further developing monocyte-based products as unwanted inflammatory signaling at the injection site or peripheral blood circulation will be attenuated.

**Abstract:**

Ex vivo culture conditions during the manufacturing process impact the therapeutic effect of cell-based products. Mimicking blood flow during ex vivo culture of monocytes has beneficial effects by preserving their migratory ability. However, the effects of shear flow on the inflammatory response have not been studied so far. Hence, the present study investigates the effects of shear flow on both blood-derived naïve and activated monocytes. The activation of monocytes was experimentally induced by granulocyte-macrophage colony-stimulating factor (GM-CSF), which acts as a pro-survival and growth factor on monocytes with a potential role in inflammation. Monocytes were cultured under dynamic (=shear flow) or static conditions while preventing monocytes’ adherence by using cell-repellent surfaces to avoid adhesion-induced differentiation. After cultivation (40 h), cell size, viability, and cytokine secretion were evaluated, and the cells were further applied to functional tests on their migratory capacity, adherence, and metabolic activity. Our results demonstrate that the application of shear flow resulted in a decreased pro-inflammatory signaling concurrent with increased secretion of the anti-inflammatory cytokine IL-10 and increased migratory capacity. These features may improve the efficacy of monocyte-based therapeutic products as both the unwanted inflammatory signaling in blood circulation and the loss of migratory ability will be prevented.

## 1. Introduction

Peripheral blood monocytes play a crucial role in producing inflammatory mediators and regulating innate and adaptive immune responses. They are recruited from peripheral blood circulation into mucosal tissues or sites of inflammation, followed by differentiation into monocyte-derived macrophages or monocyte-derived dendritic cells [1,2]. The migration of circulating monocytes occurs constitutively in non-inflamed tissues but is substantially enhanced during inflammation as they are attracted by pro-inflammatory mediators such as chemokines, complement components, and products of tissue matrix degradation. In the inflamed tissue, monocytes themselves can gain the ability to produce and secrete inflammatory mediators and exhibit pro-inflammatory and later anti-inflammatory functions [2]. In humans, three circulating monocyte subsets are classified based on relative expression levels of cell surface proteins CD14 and CD16, namely classical (CD14^+^, CD16^-^), intermediate (CD14^+^, CD16^+^), and non-classical monocytes (CD14^+^, CD16^++^) [3,4,5]. Classical monocytes comprise 80–90% of circulating monocytes and are able to transmigrate through the endothelium and enter the tissue concurrent with highly phagocytic activity. In non-lymphoid tissues, e.g., the intestine, the presence of microbial pathogens induces the differentiation of classical monocytes into either inflammatory macrophages promoting further inflammation or pro-resolving macrophages. In particular, the latter promotes tissue repair followed by a return to homeostasis dependent on the nature of the activation signal and microenvironment [2]. In contrast, the non-classical monocytes (2–11% of peripheral monocytes) are assumed to patrol the blood vessel walls and mediate endothelial cell-supporting functions as well as anti-viral responses [4,5]. The intermediate monocyte population comprises 2–8% of circulating monocytes and includes functions such as the production of reactive oxygen species, antigen presentation, T cell stimulation, and inflammatory responses. According to their migratory ability and specific mode of action, classical monocytes are of rising interest for their application as cellular-based therapeutic products to treat inflammation-related diseases or cancer [6,7,8,9,10,11]. The underlying mechanism of action is that the injected genetically or biochemically modified monocytes are recruited to the site of inflammation, followed by migration into inflamed tissue and exerting their intended therapeutic effect. However, ex vivo culture conditions during the manufacturing process of the monocytic-based product may have detrimental effects on therapeutic efficacy. There are indications that, under static conditions, the adhesion of monocytes to plastic surfaces is associated with a significant loss of migratory capacity, which might impair the homing to inflamed tissue in vivo. Indeed, the mimicking of blood flow by using an orbital shaker during ex vivo culture preserved the migratory ability of human monocytes [12]. However, the effects of shear flow on the inflammatory response of isolated monocytes have not been studied so far. Hence, the present study investigates the effect of shear flow on the cytokine release of blood-derived human naïve and activated monocytes. In addition, the viability and functional properties such as migratory capacity, adherence, and metabolic activity is assessed. The activation of monocytes was experimentally induced by the granulocyte-macrophage colony-stimulating factor (GM-CSF). GM-CSF is described as a pro-survival and activating factor on mature myeloid cells such as monocytes [13,14]. Thereby, GM-CSF conveys both pro-inflammatory effects [13,15] and anti-inflammatory and regulatory functions [6,15,16] dependent on applied dose and incubation time. In our experiment, the adherence of monocytes was prevented under both static and dynamic conditions by cell-repellent surfaces to avoid the adhesion-induced differentiation to macrophages during culture [17]. 

## 2. Materials and Methods

### 2.1. Human Monocytes

Buffy coat (BC) samples from healthy donors were provided by the Department of Transfusion Medicine, Rostock University Medical Center, Rostock, after a written consent for scientific use (approval number: A2011-140, Rostock University Ethics committee). Sodium citrate was used as an anticoagulant during blood sampling. Peripheral blood monocytes were isolated via negative selection from BCs (30–50 mL) no more than 6 h old, using the EasySepTM Direct Human Monocyte Isolation Kit (STEMCELL, Vancouver, BC, Canada). EDTA was added to the BC sample to a final concentration of 3 mM, followed by the isolation protocol according to the manufacturer’s instructions. After isolation, CD14^+^/CD16^-^ monocytes were collected from the isolation buffer by centrifugation at 300× *g* for 5 min at room temperature. The supernatant was decanted, and the pellet diluted in RPMI 1640 (specification: very low endotoxin), supplemented with 2 mM stable glutamine, 10% fetal bovine serum, 100 U/mL penicillin, and 0.1 mg/mL streptomycin (all: PAN-Biotech, Aidenbach, Germany). The purity of classical monocytes (CD14^+^/CD16^-^) ranged from 86 to 90% within the isolated lymphocytes population, as indicated by the manufacturer and confirmed by own FACS analysis (BD FACSVerse™, Biosciences Corp, Piscataway, NJ, USA). Monocytes were seeded into multiwell plates (Greiner Bio-One, Leipzig, Germany) with a concentration of 1 × 10^6^ cells/mL and were incubated at 37 °C and 5% CO_2_ according to the applied experimental design.

### 2.2. Experimental Design

**Figure 1:**
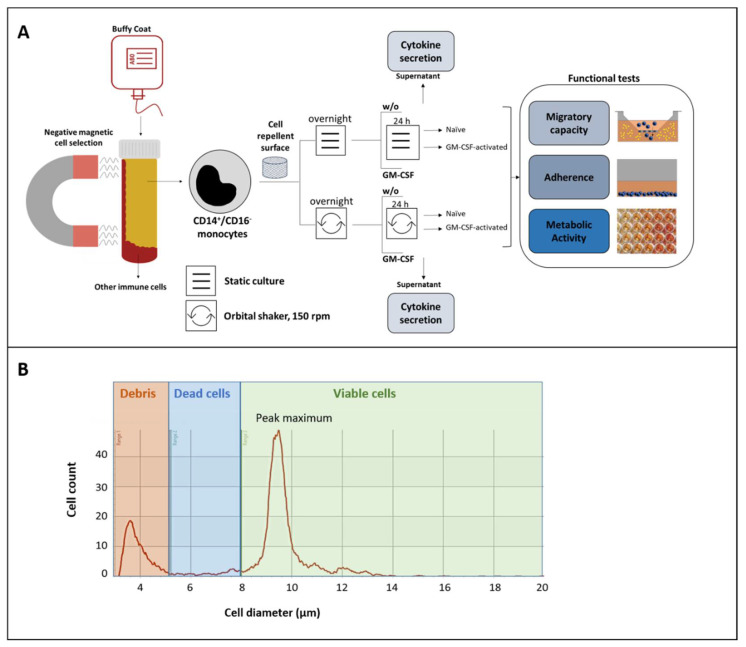
Evaluation of shear flow-mediated effects on non-adherent classical monocytes. (**A**) After isolation, CD14^+^/CD16^-^ monocytes were cultured under dynamic (=shear flow) or static conditions with or without (*w*/*o*) GM-CSF stimulation. Cytokine release was measured in cell supernatants after culture. After harvesting, naïve and GM-CSF-activated monocytes were applied to functional tests regarding their migratory capacity, adherence, and metabolic activity. (**B**) The number, size, and viability of isolated monocytes were measured directly after isolation and after culture using the CASY cell counter & analyzer. A representative result of the gated monocyte population is shown based on cell diameter, cell counts, and viability.

In all experiments on shear flow on monocytes’ functions from different donors (*n* = 20, in triplicate), cell repellent plates (Greiner Bio-One, Leipzig, Germany) were used to avoid unwanted adhesion to the plastic surface. The experimental design is illustrated in Figure 1A. After isolation, the cell number and viability were measured using the CASY cell counter (Omni Life Science, Bremen, Germany). Afterward, the monocytes were diluted in cell culture medium and incubated as a suspension culture under either static or dynamic conditions on an orbital shaker (Celltron, Infors AG, Bottmingen, Switzerland) with 150 rpm according to previously described experimental models for shear flow [12]. After an overnight resting period, monocytes were stimulated with GM-CSF for 24 h (10 ng/mL, PAN-Biotech, Aidenbach, Germany) [6] or left unstimulated using triplicates for each treatment. According to described procedures (6, 7), ex vivo culture was stopped by transferring the multiwell plates to 4 °C for 30 min. After that, non-adherent monocytes were harvested by centrifugation (300× *g* for 5 min). The cells were counted with the CASY cell counter, followed by dilution to necessary concentrations before the intended evaluation of adherence, migratory capacity, and metabolic activity. Supernatants were collected and stored at −20 °C until the measurement of cytokines. In addition to shear flow-related effects, potential impairment of functionality related to the duration of experimental procedures was evaluated. Thereby, cell size adherence, migratory ability, and metabolic rate were examined directly after isolation and compared to the results observed after overnight resting and 24 h culturing in cell culture medium.

### 2.3. Monitoring of Cell Number, Cell Size, and Viability

After cell isolation and culture, the number, size, and viability of cells were evaluated using the CASY cell counter according to the manufacturer’s instructions. To establish settings for differentiation between viable and dead cells, we used three adjusted cell suspensions (1 × 10^6^ cells/mL) and prepared them with CASY blue reagent (Omni Life Science, Bremen, Germany), followed by incubation for 2 min at room temperature. A resistance signal was generated and recorded based on cell size and conductivity, enabling the differentiation between dying/dead (low resistance signals) and living cells (high resistance signals). Accordingly, ranges for the diameter of the dead (6.00 µm–7.99 µm) and viable cells (8.00 µm–20.00 µm) were defined (Figure 1B). Cell viability was calculated as the percentage of viable cells from the total cell number (viable + dead cells). Since changes in cell size may be informative regarding the potential differentiation of monocytes, cell size was monitored by evaluating the maximum peak of cell diameter (µM).

### 2.4. Quantification of Cytokine Release

Concentrations of tumor necrosis factor-alpha (TNF-α), soluble intercellular adhesion molecule 1 (sICAM-1), interleukin 6 (IL-6), interleukin 8 (IL-8), and interleukin 10 (IL-10) were analyzed in cell culture supernatants, harvested after 24 h of stimulation with or without (w/o) GM-CSF using commercially available human enzyme-linked immunosorbent assays (ELISAs) (DuoSet^®^ ELISA Kits, R&D systems, Minneapolis, MN, USA). Analyses were performed according to the manufacturer’s instructions. 

### 2.5. Migratory Capacity

Migratory capacity was evaluated immediately after monocyte isolation and after cultivation for 24 h with or w/o GM-CSF. First, monocytes were harvested and adjusted to a final concentration of 5 × 10^6^ cells/mL. Subsequently, 100 µL of the adjusted cell suspension was seeded into 5 µm TC-Insert (Sarstedt, Nümbrecht, Germany). The inserts were placed in a 24-well culture plate (Greiner-Bio-One, Leipzig, Germany), and on the outside of the inserts, 500 µL supplemented cell culture medium with or w/o monocyte chemoattractant protein-1 (MCP-1) (25 ng/mL, R&D Systems, Minneapolis, MN, USA) as chemoattractant was added. After 2.5 h incubation at 37 °C and 5% CO_2_, the transwell chamber was removed. Subsequently, the number of cells migrated to the outside of the chamber was measured in the supernatant. The migratory capacity was expressed as the percentage of cells which migrated from the upper chamber to the lower chamber.

### 2.6. Adherence 

Monocytes were harvested directly after isolation and after 24 h stimulation with GM-CSF and adjusted to a final concentration of 1 × 10^6^ cells/mL. Subsequently, 100 µL of cell suspension were seeded per well (in triplicate) and incubated for 2.5 h at 37 °C and 5% CO_2_ under static conditions using a 96-well culture plate (Greiner-Bio-One, Leipzig, Germany). Supernatants were decanted, and adherent cells were washed with 100 µL PBS per well. Adherent cells were detached by incubation with accutase (100 µL per well) (PAN-Biotech, Aidenbach, Germany) at room temperature for 20 min. Triplicate samples were pooled in a 1.5 mL tube, and the reaction was stopped by adding the cell culture medium to a final volume of 1 mL. Monocytes were then counted using the CASY cell counter. 

### 2.7. Quantification of Metabolic Activity

Cells were harvested directly after isolation and after 24 h stimulation with GM-CSF and adjusted to a final concentration of 1 × 10^6^ cells/mL. Subsequently, 100 µL cell suspension (1 × 10^5^ cells) was transferred to each well of a 96-well culture plate in triplicates. Then, WST-1 reagent ([2-(4-Iodophenyl)-3-(4-nitrophenyl)-5-(2,4-disulfophenyl)-2H-tetrazolium], Merck, Darmstadt, Germany) was added according to the manufacturer’s instructions followed by incubation for 1.5 h at 37 °C and 5% CO_2_. After that, spectrophotometric measurement at an optical density (OD) o440/650 nm was performed on a TECAN microplate reader (Infinite m200, TECAN, Männedorf, Switzerland). The assay is based on converting the tetrazolium salt WST-1 into a colored dye by mitochondrial dehydrogenase enzymes. The results are informative for the metabolic activity of cells and used as an indirect marker for cell viability and linked to inflammatory properties of leukocytes [18].

### 2.8. Statistical Analyses

Statistical analyses were performed using SAS software, version 9.4 (SAS Institute Inc., Cary, NC, USA). The continuous response variables sICAM-1, IL-8, TNF-α, IL-6, IL-10, and metabolic activity were analyzed by Analysis of Variance (ANOVA: distribution = normal, link = identity). The response variables cell size, adherence, and migratory capacity were analyzed by a POISSON model (link = log). All data were calculated using a generalized linear mixed model (GLIMMIX procedure), including the effect of culture condition (dynamic vs. static) and treatment (GM-CSF vs. control) and their multiple interactions for the evaluation of shear flow-related effects. The general effect of ex vivo incubation on cell size and functional properties was evaluated by comparing results measured directly after cultivation and after static or dynamic culture. The random statement in the GLIMMIX procedure was used to take repeated measurements on the same donor into account. In addition, the biological sex of the blood donor was included as a random factor. Tukey–Kramer procedure was used for multiple pairwise comparisons. For the presentation of the results, the least square means (LS-means) and their standard errors (SE) were calculated and tested for each fixed effect and its interactions. An error probability of *p* < 0.05 was chosen as the limit of significance.

## 3. Results

### 3.1. Shear Flow Affects Cytokine Release and Cell Size of Non-Adherent Monocytes

**Figure 2:**
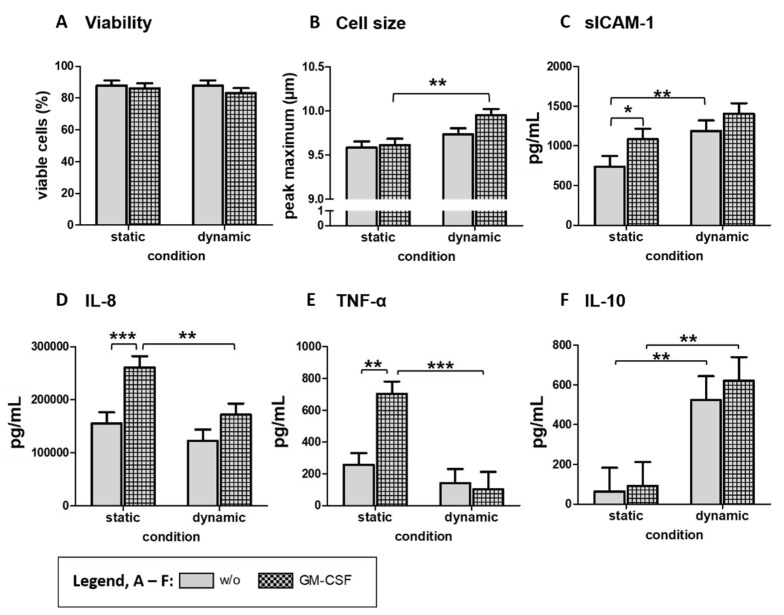
Effect of shear flow on viability, cell size, and cytokine secretion of blood-derived monocytes. After isolation from whole blood and resting, monocytes were ex vivo stimulated with GM-CSF or left unstimulated (w/o) for 24 h. Non-adherent cells were collected, and viability (**A**) and the peak maximum of cell size (**B**) were measured using the CASY cell counter. Secreted mediators or cytokines sICAM-1 (**C**), IL-8 (**D**), TNF-α (**E**), and IL-6 (**F**) were measured in cell culture supernatants by ELISA. Results are presented as LS-means + SE. Pairwise comparisons were calculated using the Tukey-Kramer test. (**A**–**F**): *n* = 14; *** *p* < 0.001, ** *p* < 0.01, * *p* < 0.05.

Monocytes were cultivated under dynamic (shear flow) or static conditions, with or without additional GM-CSF stimulation, for 24 h to evaluate the effect of shear flow. Subsequently, the cell viability and cell size were measured using the CASY cell counter. The results show that neither the stimulation with GM-CSF for 24 h nor the culture condition significantly affected the viability of monocytes in cell culture supernatants (Figure 2A). However, the monocyte’s diameter, reflected by the peak maximum, was higher after culturing under dynamic conditions with the most potent effect on GM-CSF-activated monocytes (Figure 2B). Compared to the cell size measured directly after isolation (9.51 ± 0.04 µM), GM-CSF-activated monocytes were significantly increased (*p* < 0.001). The inflammatory cytokines IL-8, and TNF-α, IL-10, and the soluble form of sICAM-1 were measured in cell culture supernatants. Interestingly, GM-CSF significantly increased secretion of sICAM-1, IL-8, and TNF-α under static but not under conditions of shear flow (Figure 2C–E). In particular, the release of TNF-α was completely prevented under dynamic conditions. In contrast, the secretion of the anti-inflammatory cytokine IL-10 was increased under dynamic conditions irrespective of GM-CSF response (Figure 2F). Furthermore, the immunomodulatory molecule sICAM-1 was significantly increased in the supernatants of unstimulated monocytes under shear flow conditions. We observed a high inter-individual variation in the response of the pleiotropic cytokine IL-6, which is why no significant effects of shear flow or GM-CSF could be calculated (Supplementary Materials Figure S1).

### 3.2. Shear Flow Affects Functional Properties of Non-Adherent Monocytes Related to Adhesion Cascade and Energy Metabolism

**Figure 3:**
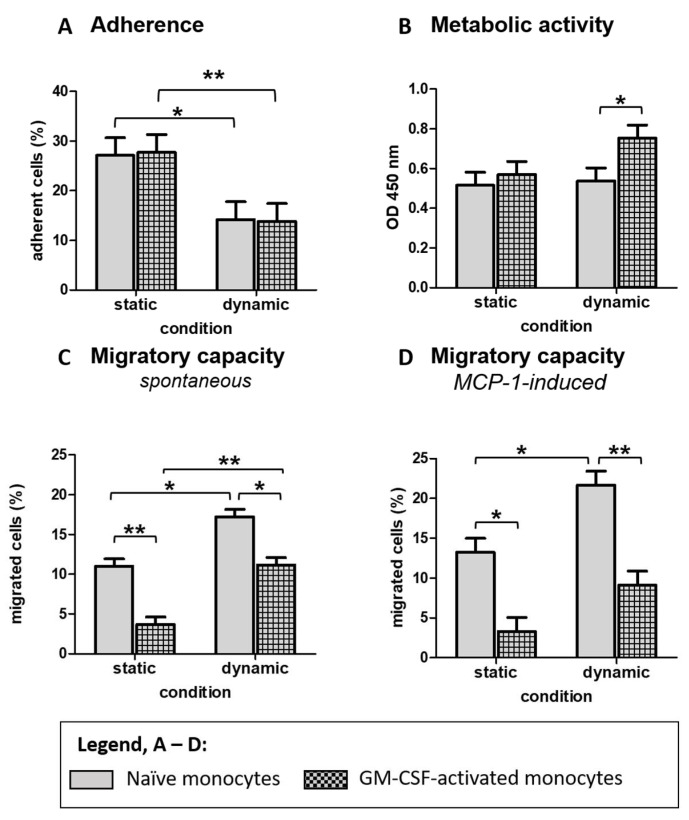
Effect of shear flow on functional properties of blood-derived monocytes. For all experiments, isolated monocytes were rested overnight and cultivated ex vivo with GM-CSF (activated) or left unstimulated (naïve) for 24 h in suspension culture. Subsequently, cells were adjusted to a concentration of 1 Mio cells/mL and then used in the respective assay. For measuring cell adhesion (**A**), monocytes were allowed to adhere to multiwell plates for 2.5 h, followed by washing with PBS. Adherent cells were enzymatically detached and counted using the CASY cell counter. The metabolic activity of cells (**B**) was examined in multiwell plates using WST-1 assay. For determining migratory capacity, monocytes were seeded into the upper chamber of a transwell insert. The lower chamber contained the monocyte medium with no cell attractants (**C**) or additional MCP-1 (**D**). Migrated cells were counted in the lower compartment after 2.5 h, and the percentage from the number of seeded cells was calculated. Results are presented as LS-means + SE. Pairwise comparisons were calculated using the Tukey-Kramer. (**A**): *n* = 8, (**B**): *n* = 6, (**C**): *n* = 6, (**D**): *n* = 11; ** *p* < 0.01, * *p* < 0.05.

Features of classical monocytes are that they can adhere to plastic surfaces or migrate through porous membranes. However, our study prevented adhesion during ex vivo stimulation with or without GM-CSF using cell culture plates with cell repellent surfaces. The cells were then harvested and transferred to dishes with an adhesive surface or transwell inserts to test the influence of the culture condition on cell adhesion and migratory capacity. Our results show that the adherence to a plastic surface was generally decreased in monocytes cultured under shear flow conditions (Figure 3A), while no effect of GM-CSF activation was detected. The metabolic activity of the cultured monocytes, measured by the reduction of tetrazolium salt to formazan, was assessed following the culture. The metabolic activity was increased in activated monocytes compared to unstimulated monocytes cultivated under shear flow conditions (Figure 3B). Interestingly, the diameter of the GM-CSF activated monocytes, grown under dynamic conditions, was the largest compared to the other conditions, which may be related to the increased metabolic activity. Moreover, the shear flow-induced reduced adherence was associated with an increased spontaneous and MCP-1-induced migration through a porous membrane, reflected by a higher percentage of migrated cells (Figure 3C,D).

### 3.3. Ex Vivo Incubation Influences Functional Properties of Blood-Derived Monocytes

**Figure 4:**
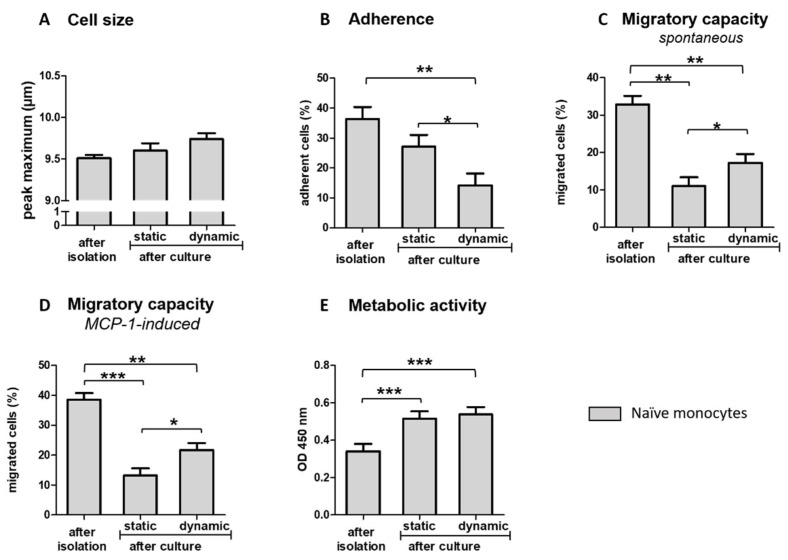
Effect of ex vivo cell incubation on blood-derived monocytes. Cell size and functional properties of naïve monocytes were measured directly after isolation from whole human blood and after static or dynamic conditions for a total of 40 h. Results are presented as LS-means + SE. Pairwise comparisons before and after cultivation were calculated using the Tukey-Kramer test. (**A**): *n* = 14, (**B**): *n* = 8, (**C**): *n* = 6, (**D**): *n* = 6; (**E**): *n* = 11; *** *p* < 0.001, ** *p* < 0.01, * *p* < 0.05.

Cell size and functional properties were assessed directly after isolation from fresh BCs and compared to results observed after the culture of naïve monocytes under dynamic or static conditions. The results are presented in Figure 4. The results reveal that the cell size of naïve monocytes tended to be increased (*p* = 0.0620, Figure 4A) while the adherence to plastic surfaces was significantly reduced after culturing under dynamic conditions compared to freshly isolated monocytes (Figure 4B). Neither dynamic nor static conditions prevent the significant loss of spontaneous or chemoattractant-induced migratory ability (Figure 4C,D). However, the loss was attenuated under shear flow conditions, as shown above by an increased migration compared to static conditions. The effect of the chemoattractant MCP-1 showed a high inter-individual variance between the BC samples. Directly after isolation, a 1.2 to 1.8-fold increase of migration in response to MCP-1 was measured in 3 of 6 donors, whereas cells of the others donors remained unaffected. Heterogeneity of chemotactic activity remained high, even after the static or dynamic culture. The metabolic activity of naïve monocytes was increased after both dynamic and static culture conditions compared to freshly isolated monocytes (Figure 4E). In contrast to GM-CSF-activated monocytes (Figure 3B), the results of naïve monocytes reveal no significant effects of culture condition on metabolic activity.

## 4. Discussion

In this study, we investigated the effects of different culture conditions on the phenotype of blood-derived classical monocytes. Monocytes were cultivated under dynamic (shear flow) or static conditions, with or without additional GM-CSF stimulation, for 24 h. The use of cell-repellent plates reliably prevented adherence in order to avoid adhesion-induced differentiation.

The viability of non-adherent monocytes, harvested from cell culture supernatants after the cultivation period, was comparable between the different culture conditions and ranged from 83 to 87%, indicating good tolerance of culture conditions. However, the monocyte’s diameter was higher after culturing under dynamic conditions, with the most substantial effect in GM-CSF-activated monocytes. Compared to the cell diameter measured directly after isolation, the data reveal a slight increase in monocytes diameter by shear flow, particularly in activated monocytes, while after static conditions, no changes were observed. Since GM-CSF acts as a growth factor [14], one may assume that GM-CSF-signaling is modulated by the presence or absence of shear flow. Interestingly, in our study, the larger GM-CSF-activated monocytes cultured under dynamic conditions exhibited the highest metabolic activity, which might be related to altered energy metabolism. Irrespective of GM-CSF activation, the metabolic activity of ex vivo cultivated monocytes was generally increased after both static and dynamic culture conditions compared to freshly isolated monocytes. According to results from tumor cell lines, an increased reduction of the tetrazolium salt WST1 was associated with increased glucose metabolism [19], indicating an increased energy demand of monocytes during suspension culture. Further studies revealed that increased glycolysis was associated with the rapid production of various metabolites that support cellular activities and cell growth [20,21,22]. Taken together, the slight increase in cell size and metabolic activity may indicate increased cell growth and metabolic alterations induced by ex vivo culture with the most substantial effect in GM-CSF-activated monocytes under shear flow conditions. In our model, we avoided the adherence of monocytes to the plate surface, and all harvested cells were non-adherent. According to the described adhesion cascade [23] and the absence of additional inflammatory stimuli such as interferon-gamma or IL-4 [6], a differentiation of GM-CSF-activated monocytes to macrophages under dynamic but not under static conditions seems unlikely.

Moreover, the shear flow-related phenotype was associated with a modulated cytokine response. Thereby, GM-CSF significantly increased the secretion of the pro-inflammatory mediators sICAM-1, IL-8, and TNF-α under static but not under shear flow conditions. In contrast, the secretion of anti-inflammatory cytokine IL-10 was generally increased by shear flow, irrespective of GM-CSF activation. These results indicate an anti-inflammatory effect of shear flow on non-adherent monocytes, which may be related to their biological function during circulation in blood. It is well accepted that under inflammatory conditions, circulating classical monocytes are rapidly recruited to the site of injury or inflammation, requiring their trafficking across the endothelium, which includes a sequence of events, a so-called adhesion cascade [24]. Assuming that shear flow mimics conditions of circulating non-adherent monocytes, reduced secretion of cytokines in response to inflammatory activation would be expected to prevent systemic and unspecific inflammatory signaling at sites of non-inflamed endothelium. Modulation of signal transduction and gene expression by biomechanical stimuli has also been described in endothelial cells in the context of atherosclerotic lesions [25]. Thereby, laminar shear stress was found to suppress the expression of pro-inflammatory genes, while under pathologic conditions, a disturbed flow enhanced the expression of pro-inflammatory genes. Therefore, we suppose that under static conditions, the loss of shear flow during culture is associated with a more pro-inflammatory phenotype in response to GM-CSF, which might be related to an activation of adhesion cascade [24,26] or beginning senescence [27]. 

Irrespective of activation with GM-CSF, the pro-inflammatory mediator sICAM-1 was significantly increased by shear flow. ICAM-1, also known as CD54, is a transmembrane protein (mICAM-1) that plays an essential role in leukocyte trafficking and several cellular immune responses [28,29]. Shedding of mICAM-1 by, e.g., proteolytic cleavage leads to the release of a soluble form of sICAM-1 (sICAM-1), which is described to interact with leucocyte function-associated antigen-1, present on the surface of T lymphocytes and activated or natural killer cells [30,31]. In our study, the increase of sICAM-1 may be related to increased shear forces under dynamic conditions, as pro-inflammatory-induced proteolytic cleavage is unlikely in the absence of pro-inflammatory mediators such as TNF-α. Interestingly, no significant effects of shear flow were found regarding the secretion of IL-6, which may be a result of the observed high inter-individual variation in cytokine response between the donors, also observed in other studies using human peripheral blood lymphocytes as a source for studying immune cell populations [32]. The potential higher variance of BC-derived human peripheral blood leukocytes compared to experiments using established cell lines should be considered to calculate the required sample number to obtain meaningful results. 

According to their biological functions, classical monocytes can adhere to different surfaces [24]. One central mechanism of monocytes for the attachment on the surface of cell culture plates is binding to charged residues on plastic via integrins αMβ2 and αXβ2 [33,34]. In our study, the ability to adhere to a plastic surface was generally decreased in monocytes cultured under shear flow conditions, while no effect of GM-CSF activation was detected. During inflammation, integrins guide monocyte migration and regulate the phenotype of macrophage differentiation [35]. Therefore, an attenuated integrin signaling would be expected to prevent enhanced emigration of circulating monocytes in non-inflamed tissue, which may be advantageous for the use of monocytes as a cellular therapeutic product. Interestingly, the shear flow-induced reduced adherence was associated with an increased spontaneous migration through a porous membrane. Our results support findings in glucocorticoid-treated human non-adherent monocytes in which a reduced adherence to plastic was associated with increased motility [36]. Compared to freshly isolated monocytes, the adherence was significantly reduced by shear flow conditions but not by static conditions. As the higher monocyte adhesion, measured directly after isolation, was associated with higher motility, we assume other mechanisms than adhesion-related integrin-signaling to be crucial for migration through the porous membrane of the transwell, which may be related to the state of cell differentiation. 

In our study, neither dynamic nor static conditions completely prevented the loss of spontaneous or chemoattractant-induced migratory ability compared to freshly isolated monocytes, indicating irreversible effects by the ex vivo culture. However, the loss of migratory capacity was significantly reduced, which is in line with a study of monocytes cultured in polypropylene tubes, describing that the loss of diapedesis due to static culture is attenuated under shear flow conditions [12]. One explanation may be that the constant contact with the plastic surface under static conditions is sufficient to activate adhesion-related signaling cascades even when firm adherence is prevented. The increased inducibility of cytokine secretion supports the potential monocyte activation in response to GM-CSF under static conditions indicating a conversion of the monocyte phenotype. Moreover, under both dynamic and static conditions, the migratory ability was compromised in GM-CSF-activated compared to naïve monocytes, which might result from the prolonged incubation with GM-CSF. However, in an additional experiment, a shortened incubation period of 16 h could completely prevent the loss of migratory ability (supplementary Figure S2), indicating that more prolonged incubation with GM-CSF might promote differentiation of monocytes accompanied by altered functional properties. 

This study investigated classical monocytes, which represent the most abundant subpopulation of circulating monocytes. Since non-classical monocytes or intermediate monocytes mediate other functions [4], the response to shear flow or GM-CSF may differ from classical monocytes. Depending on the subpopulation to be intended for cellular therapy, potential differences should be considered.

## 5. Conclusions

Our results indicate that mimicking blood flow in monocytes during ex vivo incubation is associated with the expression of a distinct monocyte phenotype characterized by reduced pro-inflammatory cytokine response to GM-CSF and reduced adherence. Concurrently, shear flow generally increased the anti-inflammatory cytokine IL-10 and migratory capacity. Taken together, we assume that shear flow can induce or maintain anti-inflammatory signaling. These features may improve the efficacy of monocyte-based therapeutic products as both the unwanted inflammatory signaling at the injection site or in blood circulation will be prevented, and the preservation of migratory ability will be improved under ex vivo culture. 

## Data Availability

Data are contained within the article.

## Supplementary Materials

The following are available online at https://www.mdpi.com/article/10.3390/biology10080748/s1, Supplementary Material: Figures S1 and S2. Migratory capacity of naïve and GM-CSF activated monocytes after a shortened incubation period.
