# Supplementary material for: Mimicking of Blood Flow Results in a Distinct Functional Phenotype in Human Non-Adherent Classical Monocytes
Source: Biology (Basel). 2021 Aug 4;10(8):748. doi: 10.3390/biology10080748 (PMC8389597; doi:10.3390/biology10080748)
Supplement: Supplementary file 1 [file biology-10-00748-s001.zip › biology-1312410-supplementary.pdf]

Supplementary Material

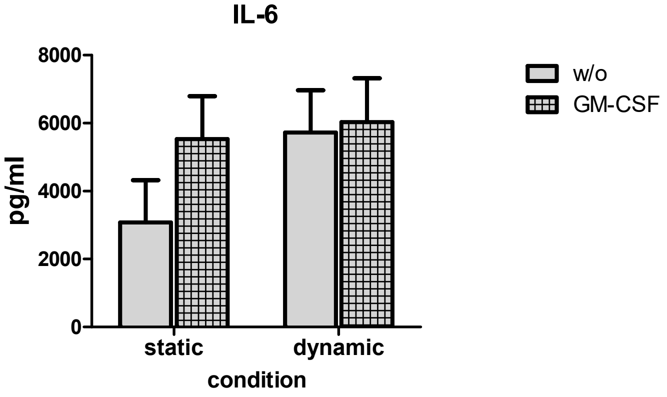

Supplementary Figure S1. Effect of shear flow on IL-6 secretion by blood-derived monocytes. After isolation from whole blood and overnight-resting, monocytes were *ex vivo* stimulated with GM-CSF or left unstimulated (w/o) for 24 h. Non-adherent cells were collected, and IL-6 was measured in cell culture supernatants by ELISA. Results are presented as LS-means + SE. Pairwise comparisons were calculated using the Tukey-Kramer test. No significant effects of shear flow or GM-CSF were calculated. n=14.

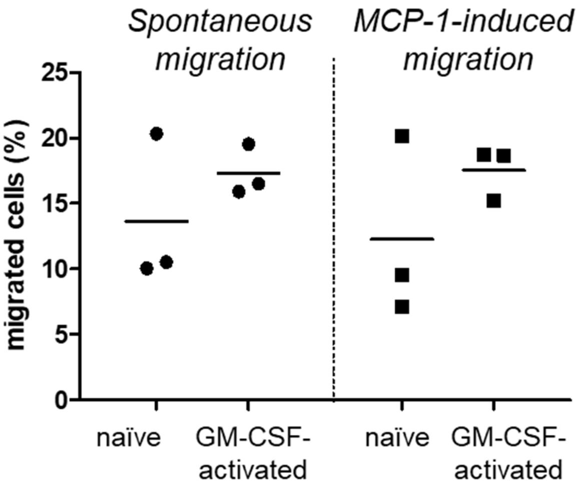

Supplementary Figure S2. Migratory capacity of naïve and GM-CSF activated monocytes after a shortened incubation period. Isolated monocytes of three BCs were cultivated *ex vivo* with GM-CSF (activated) or left unstimulated (naïve) for 16 h in suspension culture under static culture conditions. Subsequently, cells were adjusted to a concentration of 1 Mio cells/mL, followed by the evaluation of migratory capacity. The lower chamber contained the monocyte medium with no cell attractants (spontaneous) or additional MCP-1. Migrated cells were counted in the lower compartment after 2.5 h, and the percentage from the number of seeded cells was calculated.
